# Culturable Nasal Bacteria in Chronic Rhinosinusitis with Nasal Polyps: A Single-Center Observational Pilot Study

**DOI:** 10.3390/diseases14070264

**Published:** 2026-07-22

**Authors:** Camilla Laureti, Dario Benelli, Federica Zoccali, Gabriele Riccardi, Mattia Umberto Di Michele, Stefano Venarubea, Christian Barbato, Antonio Minni, Carla Petrella

**Affiliations:** 1 Division of Otolaryngology-Head and Neck Surgery, Ospedale San Camillo de Lellis, Azienda Sanitaria Locale (ASL) Rieti, Sapienza University of Rome, 02100 Rieti, Italy; camillalaureti4@gmail.com (C.L.); gabriele.riccardi@uniroma1.it (G.R.); antonio.minni@uniroma1.it (A.M.); 2 Department of Molecular Medicine, Sapienza University of Rome, 00161 Rome, Italy; dario.benelli@uniroma1.it; 3 Department of Sense Organs (DOS), Sapienza University of Rome, 00161 Rome, Italy; mattiaumberto.dimichele@uniroma1.it; 4 Clinical Pathology, San Camillo de Lellis Hospital, 02100 Rieti, Italy; 5 Institute of Biochemistry and Cell Biology (IBBC), National Research Council (CNR), 00161 Rome, Italy; 6 Interdisciplinary Department of Well-Being, Health and Environmental Sustainability (BeSSA), Sapienza University of Rome, 02100 Rieti, Italy

**Keywords:** chronic rhinosinusitis with nasal polyps, culturable bacteria, nasal swab, *Staphylococcus*, pilot study

## Abstract

Background: Bacteria colonizing the nasal cavity contribute to mucosal homeostasis, and altered sinonasal bacterial communities have been associated with chronic rhinosinusitis with nasal polyps (CRSwNP). Whether routine, culture-based microbiology can distinguish the cultivable bacterial profile of CRSwNP patients from that of controls remains unclear. Methods: In this single-center observational pilot study, middle-meatus swabs from 30 patients with CRSwNP and 30 controls were cultured on four media, and isolates were identified with the VITEK 2 system. For each species, presence or absence per subject was compared between groups using Fisher’s exact test with Benjamini–Hochberg correction for multiple comparisons; the number of cultivable species per subject was compared with the Mann–Whitney test. Results: The two groups were comparable in age and sex. Patients yielded more cultivable bacterial species per subject than controls (mean 4.4 vs. 2.9; Mann–Whitney *p* < 0.001). Several species were numerically more frequent in patients (e.g., *Staphylococcus hominis*, *S. lugdunensis*) and others in controls (e.g., *S. epidermidis*, *S. aureus*), but no individual species difference remained significant after correction. Conclusions: Using routine culture, CRSwNP was characterized chiefly by a higher number of cultivable bacterial species, whereas individual species differences were not statistically robust. These hypothesis-generating findings warrant confirmation in larger, sequencing-based studies.

## 1. Introduction

The human microbiota interacts closely with the host and contributes to numerous physiological functions, most notably the development and regulation of the immune system [1,2,3]. Within this broader picture, the nasal cavity hosts a community of commensal bacteria that contributes to respiratory mucosal homeostasis, competing with potential pathogens for space and nutrients and supporting local immune defenses [4,5]. In adults, this community is dominated by Actinobacteria and Firmicutes, with recurrent genera including *Corynebacterium*, *Cutibacterium* (formerly *Propionibacterium*), *Staphylococcus*, *Streptococcus*, *Moraxella*, and *Haemophilus* [6,7,8,9]. Its composition varies between individuals, and the boundary between commensal and potential pathogen is often difficult to draw [10]. Chronic rhinosinusitis with nasal polyps (CRSwNP) is a multifactorial disorder defined by inflammation of the nasal and paranasal sinus mucosa lasting at least 12 weeks, together with the presence of nasal polyps [11,12]. It arises from the interplay between genetic susceptibility and environmental factors, including cigarette smoke, which can alter the sinonasal bacterial community, favor bacterial adhesion and biofilm formation, and contribute to a chronic, predominantly type 2 inflammatory response [13,14,15]. Among the bacteria implicated, *Staphylococcus aureus* has been the most studied through its production of superantigens and its capacity to form biofilms [16,17]. Most current knowledge of the sinonasal bacterial community in CRSwNP derives from culture-independent, sequencing-based studies [18], whereas routine clinical microbiology, based on culture and biochemical identification, remains widely available, inexpensive, and already embedded in daily practice. Beyond its accessibility, culture offers something that culture-independent methods do not: it yields viable isolates that can be stored, characterized phenotypically, and tested for antimicrobial susceptibility or for virulence traits such as biofilm formation, all of which are directly relevant to how CRSwNP is managed in practice. This is a meaningful complement to sequencing-based work, which has emphasized a loss of bacterial diversity and community imbalance in chronic rhinosinusitis [18] but does not, on its own, provide an organism on which such downstream testing can be performed. At the same time, culture recovers only the fraction of the community able to grow under the chosen conditions and cannot quantify diversity in the sense used by sequencing, so the two strategies are best regarded as complementary. CRSwNP is, moreover, common, often recalcitrant to medical and surgical treatment, and associated with a considerable symptom burden and reduced quality of life [11,12], so a reliable account of which organisms can be recovered from such patients by everyday laboratory methods has direct practical value. Establishing whether standard, inexpensive, and widely available procedures can, by themselves, distinguish patients with CRSwNP from control subjects is therefore a necessary preliminary step and one that has received comparatively little attention before more resource-intensive molecular characterization is deployed at scale. Whether such routine, culture-based microbiology can distinguish the culturable bacterial profile of patients with CRSwNP from that of control subjects has been little investigated. In this single-center observational pilot study, we used standard nasal swab sampling, aerobic and selective culture, and VITEK 2 identification to compare patients with CRSwNP and control subjects, considering both which species were isolated and the number of culturable species detected per subject. Because culture recovers only the cultivable fraction of the bacterial community, the study is intended as a hypothesis-generating exploration rather than a comprehensive characterization of the nasal microbiota.

## 2. Materials and Methods

### 2.1. Study Design and Participants

This single-center observational pilot study was conducted between November 2023 and July 2024 at the San Camillo de Lellis Hospital in Rieti, Italy (Sapienza University of Rome), in collaboration with the Otorhinolaryngology Unit and the hospital’s Analysis Laboratory. Thirty adult patients with CRSwNP were enrolled (19 men and 11 women; mean age 43.4 ± 15.2 years); the diagnosis was confirmed according to current guidelines for sinonasal polyposis and supported by computed tomography (CT) or magnetic resonance imaging (MRI). The control group comprised thirty subjects (18 men and 12 women; mean age 40.9 ± 14.8 years) attending the same Otorhinolaryngology Unit for complaints not involving the nasal cavity and without CRSwNP; controls were not recruited as healthy volunteers, and this comparison group is therefore best described as subjects without sinonasal polyposis rather than as healthy individuals. The two groups were comparable in age and sex. Inclusion criteria were adult age (over 18 years); for patients, a confirmed diagnosis of CRSwNP based on guidelines together with CT or MRI findings; and, for all participants, no cortisone and/or antibiotic therapy for at least 15 days before enrollment. This 15-day washout was intended to limit the acute effects of recent antimicrobial or corticosteroid exposure on the cultivable bacterial community while remaining practical in an observational outpatient setting; we acknowledge that it may be shorter than ideal, particularly for corticosteroids. The study was conducted in accordance with the Declaration of Helsinki and approved by the Ethics Committee of the Policlinico Umberto I Hospital (reference 6536); informed consent was obtained from all participants.

### 2.2. Clinical Data and SNOT-22

Each participant completed a questionnaire recording demographic and clinical data, including age, sex, body mass index (BMI), smoking status, and associated conditions. Patients additionally completed the Sino-Nasal Outcome Test-22 (SNOT-22), a validated patient-reported questionnaire of 22 items, each scored from 0 (no problem) to 5 (worst possible problem), yielding a total score from 0 to 110, with higher scores indicating greater symptom burden [19]. All data were anonymized before analysis.

### 2.3. Nasal Sampling and Bacterial Culture

A swab was obtained from the middle meatus of both nostrils, following the same protocol for all participants. Each sample was inoculated onto four culture media (bioMérieux Italia, Bagno a Ripoli, Firenze, Italy): Columbia blood agar with 5% sheep blood (COS), chocolate agar with PolyViteX (PVX), mannitol salt agar (MSA2), and MacConkey agar (MCK). These media were selected to maximize recovery of both commensal and potentially pathogenic organisms: COS as a non-selective medium, PVX for fastidious organisms such as *Haemophilus* and *Neisseria*, MSA2 as a selective medium for staphylococci, and MCK as a selective medium for Gram-negative bacteria. COS and PVX were incubated both aerobically and anaerobically, while MSA2 (selective for staphylococci) and MCK (selective for Gram-negative bacteria) were incubated aerobically at 37 °C. After 48 h, the plates were examined, and morphologically distinct colonies were subcultured; after a further 24 h, the colonies of interest were examined microscopically following Gram staining [20]. Biochemical identification was performed with the VITEK 2 system (bioMérieux Italia, Bagno a Ripoli, Firenze, Italy): each isolate was suspended in saline, adjusted to the appropriate McFarland turbidity standard, and processed using the corresponding identification cards [21].

### 2.4. Statistical Analysis

For each subject, every species was recorded as present or absent. For each species, the prevalence (proportion of positive subjects) was compared between patients and controls using Fisher’s exact test, and *p* values were corrected for multiple comparisons with the Benjamini–Hochberg false discovery rate (FDR), reported as q values; effect sizes were expressed as odds ratios with 95% confidence intervals, using the Haldane–Anscombe correction for species absent in one group. The number of culturable species per subject was compared between groups with the Mann–Whitney U test. In addition to the analysis of all species, a focused analysis was performed on the staphylococci most consistently implicated in CRSwNP in the literature (*Staphylococcus epidermidis*, *S. aureus*, *S. hominis*, and *S. lugdunensis*), with Benjamini–Hochberg correction applied within this restricted set. Age was compared with Welch’s *t*-test and sex with Fisher’s exact test. Tests were two-sided, with *p* < 0.05 (and FDR q < 0.05 for the per-species comparisons) considered statistically significant. Given the exploratory nature of this pilot study, the individual species comparisons are reported as descriptive and hypothesis-generating. No formal a priori sample-size or power calculation was performed, consistent with the exploratory, hypothesis-generating nature of this single-center pilot study. Analyses were performed in R version 4.3 (R Foundation for Statistical Computing, Vienna, Austria).

## 3. Results

### 3.1. Study Population

Thirty patients with CRSwNP and thirty control subjects were included. The two groups were comparable for age (43.4 ± 15.2 vs. 40.9 ± 14.8 years; *p* = 0.52) and sex (19 of 30 vs. 18 of 30 men; *p* = 1.00; Table 1). Within the patient group, the mean BMI was 25.2 ± 3.3 kg/m^2^, and the mean SNOT-22 score was 30.9 ± 15.7 (range 9 to 71); BMI and SNOT-22 were not systematically recorded for the control group. Current smoking status, available for both groups, is reported in Table 1.

### 3.2. Number of Culturable Species per Subject

**Figure 1:**
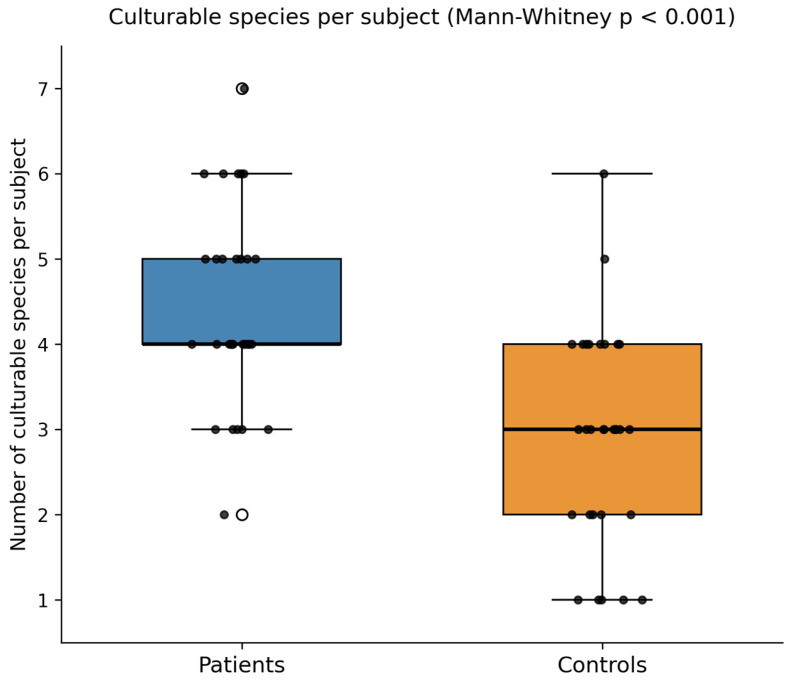
Number of culturable bacterial species isolated per subject in patients with chronic rhinosinusitis with nasal polyps (CRSwNP, *n* = 30) and controls (*n* = 30). Boxes show the median and interquartile range, and whiskers extend to the most extreme values within 1.5 times the interquartile range; individual subjects are shown as overlaid points. Patients harbored a higher number of culturable species than controls (median 4 vs. 3; Mann–Whitney U test, *p* < 0.001).

Patients yielded a higher number of culturable bacterial species per subject than controls (mean 4.4, median 4, interquartile range 4 to 5, range 2 to 7, versus mean 2.9, median 3, interquartile range 2 to 4, range 1 to 6). This difference was statistically significant (Mann–Whitney U test, *p* < 0.001) and is shown in Figure 1.

### 3.3. Species-Level Comparison

**Figure 2:**
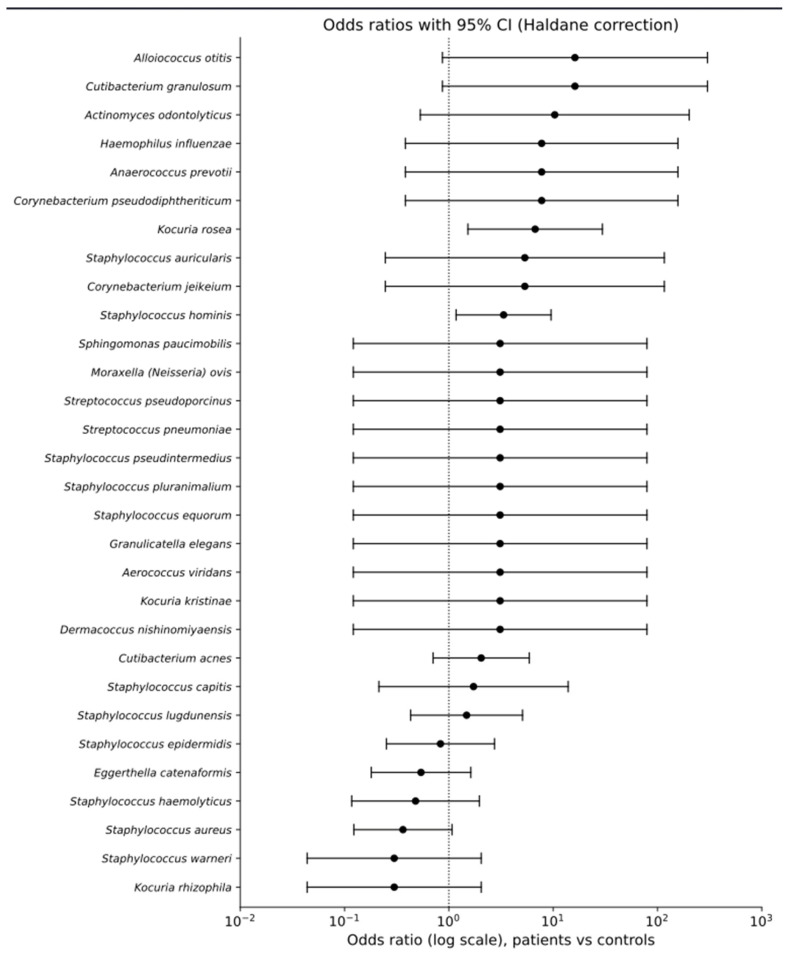
Odds ratios for the presence of each culturable bacterial species in patients with CRSwNP versus controls, with 95% confidence intervals (Haldane–Anscombe correction applied for species absent in one group). Each point represents the odds ratio for one species, ordered by odds ratio, and the horizontal lines represent the corresponding 95% confidence intervals; the dotted vertical line marks an odds ratio of 1 (no association), and the *x*-axis is on a logarithmic scale. Points to the right of the line indicate species more frequently isolated in patients; points to the left indicate species more frequent in controls. Wide confidence intervals reflect the small sample size and the low prevalence of many species. After Benjamini–Hochberg correction for multiple comparisons, none of the species differences was statistically significant.

Across the whole cohort, 30 species were identified. *Staphylococcus epidermidis* was the most frequently isolated species in both groups, although it was somewhat less frequent in patients than in controls (77% vs. 80%). Several species were numerically more frequent in patients, including *Staphylococcus hominis* (60% vs. 30%), *Cutibacterium acnes* (43% vs. 27%), *Kocuria rosea* (37% vs. 7%), and *Staphylococcus lugdunensis* (23% vs. 17%); conversely, *Staphylococcus aureus* (23% vs. 47%) and *Eggerthella catenaformis* (23% vs. 37%) were more frequent in controls. A number of species were detected only in patients, most notably *Alloiococcus otitis* and *Cutibacterium granulosum* (each 20%), *Actinomyces odontolyticus* (13%), and *Haemophilus influenzae* and *Anaerococcus prevotii* (each 10%). After correction for multiple comparisons, however, none of these individual species differences remained statistically significant (all FDR q > 0.05; lowest q ≈ 0.24). A focused analysis restricted to the staphylococci most often implicated in CRSwNP (*S. epidermidis*, *S. aureus*, *S. hominis*, and *S. lugdunensis*) gave the same picture: *S. hominis* showed the lowest uncorrected *p* value (0.037), but no species in this set reached statistical significance after correction. The odds ratios with 95% confidence intervals for all species are shown in Figure 2, and the corresponding prevalences are reported in Table 2.

## 4. Discussion

In this culture-based pilot study, the clearest difference between patients with CRSwNP and controls was quantitative: patients yielded a higher number of culturable bacterial species per subject. This was the only comparison that remained robust, and it suggests that routine clinical microbiology can capture a measurable difference in the cultivable bacterial community associated with CRSwNP. It should be noted that culture-independent, sequencing-based studies have generally described reduced bacterial diversity, or even community collapse, in chronic rhinosinusitis [18]; the higher number of culturable species observed here in patients is therefore not equivalent to greater microbial diversity. This apparent contrast underlines that culture-based counts and sequencing-based diversity capture different facets of the bacterial community and should not be conflated. What a higher number of culturable species represents biologically remains to be established: it may reflect reduced colonization resistance, a more permissive or altered mucosal environment, or greater instability of the bacterial community in CRSwNP, rather than a genuinely richer community in the ecological sense. Several non-exclusive mechanisms could, in principle, underlie a greater number of culturable organisms in the diseased mucosa. Chronic type 2 inflammation, epithelial barrier disruption, altered mucus composition, and impaired mucociliary clearance may create a more permissive niche in which a broader range of organisms can establish and be recovered by culture; a relative loss of dominant protective commensals, such as coagulase-negative staphylococci with barrier-supporting properties, could in turn lower colonization resistance and allow additional species to co-exist [4,5,22,23]. Whether the higher culturable count reflects any of these processes or is instead influenced by sampling and culture conditions cannot be resolved by a cross-sectional, culture-based design, and we therefore present this observation strictly as a starting point for mechanistic, longitudinal studies. At the level of individual species, several descriptive differences were apparent, but none survived correction for multiple comparisons, and they should be regarded as hypothesis-generating. *Staphylococcus epidermidis*, the most frequently isolated commensal in both groups, tended to be less frequent in patients; this coagulase-negative staphylococcus has been credited with immunomodulatory and barrier-supporting functions in the nasal mucosa [22,23], so a relative reduction, if confirmed, could be biologically relevant. *Staphylococcus aureus*, classically implicated in CRSwNP through superantigen production and biofilm formation [16,24,25], was likewise less frequently isolated in patients than in controls, a finding to interpret cautiously, since culture does not capture biofilm-associated or low-abundance organisms. Conversely, *Staphylococcus hominis* and *Staphylococcus lugdunensis* were numerically more frequent in patients; *S. lugdunensis* in particular has attracted growing interest as a nasal coagulase-negative staphylococcus with both commensal and potentially pathogenic features [26,27]. These observations are consistent with the literature but, in our data, remain trends rather than established associations. Notably, even when the analysis was restricted to the staphylococci most strongly implicated in the literature, no individual species difference was statistically significant. Within patients, exploratory analyses showed that neither smoking status nor SNOT-22 symptom burden was associated with the number of culturable species (Supplementary Table S1), consistent with the descriptive, hypothesis-generating nature of these findings. The choice of a culture-based approach deserves explicit consideration in relation to culture-independent molecular methods. Routine culture is inexpensive, widely available, and recovers viable organisms that can be identified to the species level and used for downstream phenotypic or antimicrobial testing; however, it captures only the fraction of the community able to grow under the chosen conditions and underestimates anaerobic, fastidious, and low-abundance taxa. Culture-independent methods, such as 16S rRNA gene sequencing and shotgun metagenomics, access this hidden fraction and allow a formal assessment of microbial diversity and, for metagenomics, of functional potential and are now the mainstay for characterizing human-associated microbial communities [28,29]; in the sinonasal tract specifically, sequencing-based surveys have described the bacterial community of chronic rhinosinusitis in this way [18]. These methods carry their own limitations, including sensitivity to DNA extraction and primer choice, detection of DNA from non-viable organisms, and a particular vulnerability to contamination in low-biomass samples such as nasal swabs. The two strategies are therefore complementary rather than interchangeable: culture reports which organisms are alive and recoverable, whereas sequencing reports which are present. In practical terms, this complementarity has direct implications for CRSwNP. Sequencing can map the breadth of the community and flag a loss of diversity, but it does not, by itself, yield an isolate on which to test antimicrobial susceptibility or to interrogate virulence traits such as biofilm formation, features that are central to the pathogenic role attributed to *Staphylococcus aureus* in this disease [16,24,25]. Culture, conversely, delivers exactly such isolates but underestimates the fastidious, anaerobic, and low-abundance members that sequencing detects. A pragmatic reading of our findings is therefore that routine culture can serve as an inexpensive first-line screen able to flag a quantitative difference between patients and controls, to be followed, in adequately resourced studies, by paired sequencing and susceptibility testing that together reconstruct both the composition and the functional and therapeutic relevance of the sinonasal community. From a translational standpoint, a low-cost screening step that can be performed in any clinical microbiology laboratory may be useful for stratifying which patients warrant more detailed molecular investigation and for generating hypotheses that can then be tested prospectively; it may also facilitate multicenter collaboration, since standardized culture is more readily harmonized across laboratories than sequencing pipelines, which remain sensitive to differences in extraction, amplification, and bioinformatic processing. We would stress, however, that none of these applications follow automatically from the present data: our findings establish that a difference exists and is measurable by simple means but not what it signifies, and the necessary next step is a larger, prospectively designed study that pairs culture with sequencing and links both to detailed clinical phenotyping. Importantly, concepts such as microbial diversity and dysbiosis are defined within these comprehensive, diversity- and function-based frameworks, and for this reason, we have deliberately avoided applying them to our culture-based data [30,31]. In the present study, molecular characterization was not feasible because no samples had been cryopreserved; our findings should accordingly be read as describing the culturable bacterial profile, and the most informative next step would be the prospective collection and storage of samples for paired culture and sequencing analyses. Several limitations must be emphasized. First, the sample was small (30 per group), which limited statistical power: apart from the number of species per subject, no individual comparison reached significance after correction, and the findings should be considered exploratory. Second, controls were recruited among subjects attending the same ENT unit for non-nasal complaints rather than as healthy volunteers, which may have attenuated the differences between groups. Third, BMI was not available for controls, so residual confounding by unmeasured factors cannot be excluded; the 15-day washout from antibiotics and corticosteroids, although applied uniformly, may also be shorter than ideal. In addition, because culture has limited sensitivity for low-abundance and fastidious organisms, species detected only in patients should be interpreted with caution, as they may reflect non-recovery in controls rather than true absence. Finally, this was a single-center study. Within these constraints, our data indicate that routine culture can reveal a reproducible quantitative difference between CRSwNP patients and controls and can generate testable, species-level hypotheses; the direction of any causal relationship remains to be established.

## 5. Conclusions

In this single-center pilot study, routine culture-based microbiology distinguished patients with CRSwNP from controls chiefly by a higher number of culturable bacterial species per subject, whereas differences in individual species, although several were apparent, did not survive correction for multiple comparisons. These findings should be regarded as hypothesis-generating: they indicate that an inexpensive and widely available method can detect a reproducible quantitative difference associated with CRSwNP, but they do not support conclusions about specific pathogenic species or about microbial diversity in the culture-independent sense. Larger, multicenter studies combining culture with sequencing-based methods and antimicrobial susceptibility testing of recovered isolates and including better-characterized control groups will be needed to confirm and extend these observations.

## Figures and Tables

**Table 1:** Characteristics of the subjects included in the study: 30 patients with chronic rhinosinusitis with nasal polyps (CRSwNP) and 30 controls. Values are expressed as mean ± standard deviation or number (percentage). *p*: Welch’s *t*-test for age and Fisher’s exact test for sex and current smoking; the groups did not differ for any of these variables. SD = standard deviation.

Characteristic	Patients (*n* = 30)	Controls (*n* = 30)	*p*
Age, years (mean ± SD)	43.4 ± 15.2	40.9 ± 14.8	0.52
Male sex, *n* (%)	19 (63.3)	18 (60.0)	1.00
Current smokers, *n* (%)	12 (40.0)	8 (26.7)	0.41

**Table 2:** Prevalence of culturable bacterial species in patients with chronic rhinosinusitis with nasal polyps (CRSwNP, *n* = 30) and controls (*n* = 30), grouped by phylum. Values are the number of positive subjects (percentage). *p*, Fisher’s exact test; q, Benjamini–Hochberg false discovery rate. After correction for multiple comparisons, no species differed significantly between the two groups (all q > 0.05).

Species	Patients, *n* (%)	Controls, *n* (%)	*p*	q (FDR)
**Actinobacteria**				
*Cutibacterium acnes*	13 (43.3)	8 (26.7)	0.279	0.837
*Kocuria rosea*	11 (36.7)	2 (6.7)	0.010	0.237
*Cutibacterium granulosum*	6 (20.0)	0 (0.0)	0.024	0.237
*Actinomyces odontolyticus*	4 (13.3)	0 (0.0)	0.112	0.562
*Corynebacterium jeikeium*	2 (6.7)	0 (0.0)	0.492	0.922
*Corynebacterium pseudodiphtheriticum*	3 (10)	0 (0.0)	0.237	0.791
*Dermacoccus nishinomiyaensis*	1 (3.3)	0 (0.0)	1.000	1.000
*Kocuria kristinae*	1 (3.3)	0 (0.0)	1.000	1.000
*Kocuria rhizophila*	1 (3.3)	4 (13.3)	0.353	0.883
**Firmicutes**				
*Staphylococcus epidermidis*	23 (76.7)	24 (80.0)	1.000	1.000
*Staphylococcus hominis*	18 (60.0)	9 (30.0)	0.037	0.277
*Eggerthella catenaformis*	7 (23.3)	11 (36.7)	0.399	0.920
*Staphylococcus aureus*	7 (23.3)	14 (46.7)	0.103	0.562
*Staphylococcus lugdunensis*	7 (23.3)	5 (16.7)	0.748	1.000
*Alloiococcus otitis*	6 (20.0)	0 (0.0)	0.024	0.237
*Anaerococcus prevotii*	3 (10.0)	0 (0.0)	0.237	0.791
*Staphylococcus haemolyticus*	3 (10.0)	6 (20.0)	0.472	0.922
*Staphylococcus auricularis*	2 (6.7)	0 (0.0)	0.492	0.922
*Staphylococcus capitis*	2 (6.7)	1 (3.3)	1.000	1.000
*Aerococcus viridans*	1 (3.3)	0 (0.0)	1.000	1.000
*Granulicatella elegans*	1 (3.3)	0 (0.0)	1.000	1.000
*Staphylococcus equorum*	1 (3.3)	0 (0.0)	1.000	1.000
*Staphylococcus pluranimalium*	1 (3.3)	0 (0.0)	1.000	1.000
*Staphylococcus pseudintermedius*	1 (3.3)	0 (0.0)	1.000	1.000
*Staphylococcus warneri*	1 (3.3)	4 (13.3)	0.353	0.883
*Streptococcus pneumoniae*	1 (3.3)	0 (0.0)	1.000	1.000
*Streptococcus pseudoporcinus*	1 (3.3)	0 (0.0)	1.000	1.000
**Proteobacteria**				
*Haemophilus influenzae*	3 (10.0)	0 (0.0)	0.237	0.791
*Moraxella* (*Neisseria*) *ovis*	1 (3.3)	0 (0.0)	1.000	1.000
*Sphingomonas paucimobilis*	1 (3.3)	0 (0.0)	1.000	1.000

Bold formats indicate phylum names.

## Data Availability

The datasets used and/or analyzed during the current study are available from the corresponding author upon reasonable request.

## Supplementary Materials

The following supporting information can be downloaded at https://www.mdpi.com/article/10.3390/diseases14070264/s1, Table S1: exploratory within-group analyses of the association between smoking status or SNOT-22 symptom burden and the number of culturable species per subject.

## Abbreviations

The following abbreviations are used in this manuscript:

BMI	body mass index
CI	confidence interval
COS	Columbia blood agar with 5% sheep blood
CRSwNP	chronic rhinosinusitis with nasal polyps
CT	computed tomography
ENT	ear, nose and throat
FDR	false discovery rate
MCK	MacConkey agar
MRI	magnetic resonance imaging
MSA2	mannitol salt agar
PVX	chocolate agar with PolyViteX
SD	standard deviation
SNOT-22	Sino-Nasal Outcome Test-22
