# Supplementary material for: Culturable Nasal Bacteria in Chronic Rhinosinusitis with Nasal Polyps: A Single-Center Observational Pilot Study
Source: Diseases. 2026 Jul 22;14(7):264. doi: 10.3390/diseases14070264 (PMC13409259; doi:10.3390/diseases14070264)
Supplement: Supplementary file 1 [file diseases-14-00264-s001.zip › diseases-4413647-supplementary.pdf]

Supplementary Materials

Culturable nasal bacteria in chronic rhinosinusitis with nasal polyps: a single-center observational pilot study

As exploratory, hypothesis-generating analyses, we assessed within each group whether the number of culturable species per subject was related to smoking status, and, in patients, whether it correlated with the SNOT-22 total score (available only in patients). These analyses are reported here in full and summarized in the main-text Discussion. Given the small subgroups, they should be interpreted with caution and were not used to draw inferential conclusions.

Table S1. Exploratory within-group analyses of the association between smoking status or SNOT-22 symptom burden and the number of culturable bacterial species per subject.

Analysis	Groups / measure	Test	p
Culturable species per subject, patients: smokers vs non-smokers	12 vs 18 subjects; median 4 vs 4 (mean 4.3 vs 4.5)	Mann-Whitney U	0.68
Culturable species per subject, controls: current smokers vs non-smokers	8 vs 22 subjects; mean 3.25 vs 2.82	Mann-Whitney U	0.36
Culturable species per subject vs SNOT-22 total score, patients	n = 30; Spearman rho = 0.03	Spearman	0.86

Smoking was recorded as current smoking status. The number of culturable species per subject did not differ by smoking status in either group, and did not correlate with SNOT-22 symptom burden in patients. All tests were two-sided; analyses were performed in R version 4.3.
